# Steatotic Donor Transplant Livers: Preservation Strategies to Mitigate against Ischaemia-Reperfusion Injury

**DOI:** 10.3390/ijms25094648

**Published:** 2024-04-24

**Authors:** Syed Hussain Abbas, Carlo Domenico Lorenzo Ceresa, Joerg-Matthias Pollok

**Affiliations:** 1 Oxford Transplant Centre, Nuffield Department of Surgical Sciences, University of Oxford, Oxford OX1 2JD, UK; hussain.abbas@nds.ox.ac.uk; 2 Department of Hepatopancreatobiliary and Liver Transplant Surgery, Royal Free Hospital, Pond Street, Hampstead, London NW3 2QG, UK; carlo.ceresa1@nhs.net; 3 Division of Surgery & Interventional Science, University College London, Gower Street, London WC1E 6BT, UK

**Keywords:** organ preservation solution, normothermic machine perfusion, ischaemia-free liver transplantation, defatting

## Abstract

Liver transplantation (LT) is the only definitive treatment for end-stage liver disease, yet the UK has seen a 400% increase in liver disease-related deaths since 1970, constrained further by a critical shortage of donor organs. This shortfall has necessitated the use of extended criteria donor organs, including those with evidence of steatosis. The impact of hepatic steatosis (HS) on graft viability remains a concern, particularly for donor livers with moderate to severe steatosis which are highly sensitive to the process of ischaemia-reperfusion injury (IRI) and static cold storage (SCS) leading to poor post-transplantation outcomes. This review explores the pathophysiological predisposition of steatotic livers to IRI, the limitations of SCS, and alternative preservation strategies, including novel organ preservation solutions (OPS) and normothermic machine perfusion (NMP), to mitigate IRI and improve outcomes for steatotic donor livers. By addressing these challenges, the liver transplant community can enhance the utilisation of steatotic donor livers which is crucial in the context of the global obesity crisis and the growing need to expand the donor pool.

## 1. Introduction

Hepatic steatosis (HS) is an early manifestation of metabolic dysfunction-associated steatotic liver disease (MASLD). MASLD is the most common cause of liver disease worldwide and affects one in three of the UK population [1,2]. This is also evident in United Kingdom (UK) deceased donors; those with a Body Mass Index (BMI) of ≥30 kg/m^2^ have increased from 23% to 29% in the past decade [3]. Similarly, in the United States, it is estimated that by 2030, 48.9% of the adult population will be obese with a BMI ≥ 30 kg/m^2^ and 24.2% will be severely obese with a BMI ≥ 35 kg/m^2^ [4]. With the ongoing global obesity crises, it is inevitable that MASLD (resulting in metabolic-associated steatohepatitis, MASH) will become one of the primary indications for liver transplantation (LT) and at the same time, HS will also become more prevalent in the donor pool [5,6].

Since the advent of LT, HS has been associated with poor outcomes including early allograft dysfunction (EAD), primary non-function (PNF), and inferior graft/patient survival [7]. Despite the impact of HS on LT outcomes, the quantification of HS has been heterogenous amongst liver transplant units [8,9,10], consequently, this is also reflected in the assessment and reporting of LT outcomes [11,12,13].

In 2021, Neil et al. published the Banff consensus recommendations for reporting of donor HS into three categories [14]: (i) Large droplet Macrovesicular Steatosis (Ld-MaS), characterised by a single fat vacuole causing cellular distension, being larger than adjacent non-steatotic/minimally steatotic hepatocytes with nucleus displacement to the hepatocyte periphery; (ii) Small droplet Macrovesicular Steatosis (Sd-MaS, previously described as microvesicular steatosis), characterised by presence of fat vacuoles that are not true Ld-MaS; and (iii) True Microvesicular Steatosis, characterised by multiple tiny droplets occupying hepatocytes with a signature foam-like appearance which require a specialised ‘fat’ stain to confirm their presence. Typically, these are either non-zonal aggregates or appear with diffuse liver involvement that typically presents in the setting of acute liver failure, rather than in liver grafts retrieved for transplantation [14].

In the clinical setting of LT, Sd-MaS has been historically described as microvesicular steatosis. Sd-MaS is known to increase during preservation and at reperfusion and is a transient short-lived process that indicates both liver stress/injury and recovery/regeneration from these processes. In general, ≥30% Sd-MaS is considered to be safe with no overall impact on graft survival [14,15]. However, the presence of moderate (30–60%) or severe (≥60%) Ld-MaS is associated with reduced tolerance to static cold storage (SCS, the gold standard for organ preservation) and an increased sensitivity to the process of ischaemia-reperfusion injury (IRI), clinically manifesting as post-reperfusion syndrome (PRS), EAD, requirement for renal replacement therapy (RRT), and reduced graft/patient survival [11,12]. Ld-MaS is widely acknowledged to be a negative prognostic factor in models that predict graft/patient survival following LT [15,16]. Consequently, this reflects the reservation and caution of liver transplant units in utilising donor livers with evidence of moderate-severe steatosis [13,17,18,19,20] and in the UK, donor livers with evidence of HS account for 39% of organ discards [21]. Recently, a large study analysing data from the Scientific Registry of Transplant Recipients demonstrated that Ld-MaS ≥ 31% was associated with lower odds of donor liver utilisation and the use of such livers was associated with an increased risk of graft failure by 53% [22]. Despite efforts to standardise quantification of HS in the setting of clinical LT, the lack of uniformity in the histological assessment of HS should be considered when reporting outcomes [14].

Overall, it is increasingly evident that donors with evidence of HS will constitute a large cohort of the donor pool, compounded by high discard rates in the presence of an ever-increasing donor organ shortage. Therefore, it is necessary for the liver transplant community to focus on strategies (beyond minimisation of cold ischaemia time and low MELD recipient selection) [19,23,24] to improve the safe transplantation and outcomes of donor livers with moderate-severe steatosis to address the evolving challenges posed by the global obesity crisis. This review will discuss the pathophysiological factors that predispose steatotic livers to amplified IRI, the impact of SCS, organ preservation strategies including novel organ preservation solutions, and normothermic machine perfusion as a platform to mitigate against IRI in these high-risk livers.

## 2. Hepatic Steatosis and Ischaemia-Reperfusion Injury

### 2.1. Mechanism of Ischaemia-Reperfusion Injury

In the context of LT, IRI is a sterile inflammatory response occurring following restoration of blood supply (circulation) following a period of ischaemia. The process and cascade of IRI is initiated in the mitochondria; during ischaemia, cellular metabolism is switched to anaerobic glycolysis and in the mitochondria, the absence of oxygen results in the disruption of the electron transport chain resulting in the accumulation of succinate, reverse electron transfer with detachment of flavin mononucleotide from mitochondrial complex 1 [25,26,27]. Subsequent accumulation of lactate, compounded by adenosine triphosphate (ATP) depletion (with reliance of glycogen stores for energy generation), causes cellular acidosis and electrolyte imbalances. Following reperfusion in the recipient, the rapid restoration of oxygen results in a burst of reactive oxygen species (ROS) due to the negative potential across the mitochondrial matrix generated during cold ischaemia [28]. In addition, ROS initiate a sterile immune response characterised by the release of high-mobility group box 1 (HMGB1) and nuclear factor κβ (NF-κβ). HMGB1 and NF-κβ signalling during reperfusion results in Kupffer cell activation, neutrophil immune cell recruitment with formation of neutrophil extracellular traps (NETs), microcirculatory failure, and hepatocellular injury with cell death processes [29,30,31].

Steatotic livers do not tolerate SCS well and have increased sensitivity to the process of IRI. In the clinical setting of LT, elevated hepatocellular injury markers including transaminases are associated with histological evidence of IRI [32,33] and this is evident in recipients transplanted with steatotic livers demonstrating higher early post-operative transaminase levels and EAD [17,19,34,35,36,37,38,39,40]. Experimental models indicate that hepatocellular damage (as a result of IRI) is initiated within hepatic parenchymal cells and the presence of excess lipids within hepatocytes contribute to an amplified IRI response [41,42,43]. Evidence from a hepatocyte IRI cell culture model suggests that the degree of hepatocellular injury (quantified by transaminase release) correlates with the degree of intrahepatic triglyceride (IHTG) content and a reduction in IHTG results in reduced hepatocellular injury. These findings suggest that HS is independently associated with IRI [42].

Overall, the underpinning pathophysiological mechanisms are not completely understood but can be explained through a number of complex interlinked cellular processes and mechanisms: (i) mitochondrial oxidative stress; (ii) microcirculatory distortion and impaired sinusoidal blood flow; (iii) lipid perioxidation; (iv) pro-inflammatory environment; (v) hypoxia inducible factors (HIFs); and (vi) damage-associated molecular patterns (DAMPs) and NETs.

### 2.2. Mitochondrial Oxidative Stress

Experimental data point to a differing inflammatory response to IRI in steatotic compared to lean livers, characterised by increased mitochondrial oxidative stress and inadequate ATP restoration following SCS that increase the vulnerability of steatotic livers to IRI [43,44,45]. The deterioration in adequate mitochondrial function (necessary for cellular viability) is triggered by ROS production which results in altered energy metabolism, disrupted cellular bioenergetics and cellular function which results in cell death [46,47,48,49]. Previous studies have demonstrated that the mitochondrial proton adenosine triphosphate (ATPase) activity required for ATP synthesis and oxidative phosphorylation is rapidly reduced following 6 h of SCS in steatotic compared to lean livers [50,51,52]. The lack of ATP restoration consequently results in ATP-dependant Na^+^/K^+^ pump failure contributing to cellular swelling and necrosis (rather than apoptosis which is ATP dependant) in steatotic livers [53,54].

Furthermore, the mitochondrial uncoupling protein-2 (UCP-2) required for the regulation of proton leakage across the inner membrane is paradoxically increased in steatotic livers in an attempt to counteract oxidative stress and ROS production as well as prevent chronic hepatocellular lipid accumulation. However, due to dysregulation in ATP synthesis and diminished ATP levels, the overexpression of UCP-2 limits the capacity of hepatocytes to respond to increasing energy demands at reperfusion resulting in a mitochondrial permeability transition (MPT) and potential membrane failure [45,55].

Comparatively, in lean livers, cell death is mainly a result of apoptosis (an energy dependant process) and the ATP depletion in steatotic livers results in an inability to induce apoptosis resulting in other pathways of programmed cell death and necrosis [34,36,41,42]. This is evident in increased iron overload, capase-1, capase-9, receptor-interacting kinase 1 (RIPK1) and receptor-interacting kinase 3 (RIPK3) expression observed in steatotic hepatocytes exposed to IRI, indicating ferroptosis, pyroptosis, necroptosis, and MPT-mediated necrosis mechanisms, respectively [44,56,57,58].

### 2.3. Microcirculatory Distortion and Impaired Sinusoidal Blood Flow

Lipid droplet accumulation in steatotic livers results in structural distortion and obstruction of microcirculation and sinusoidal blood flow compared to lean livers [59,60,61,62,63]. Following graft reperfusion, impairment of microcirculation can be exacerbated by hepatocellular membrane rupture resulting in the expulsion of lipid droplets into the extracellular space (lipopeliosis) causing obstruction of liver sinusoids and further compounding the issue [64,65]. In addition, the microcirculatory distortion and impaired blood flow contribute to a chronic hypoxic state due to inadequate oxygenation. This results in exacerbated ischaemic injury upon reperfusion characterised by increased ROS production and Kupffer cell activation. The activated Kupffer cells produce endothelin-1 (ET-1, vasoconstrictor) in excess to induce nitric oxide synthase (iNOS, vasodilator). This imbalance promotes microcirculatory damage due to sinusoidal vasoconstriction (and limited blood flow) during reperfusion [63,66,67]. The consequent injury impairs the role of the endoplasmic reticulum (ER) involved in hepatocellular lipid metabolism, protein synthesis, and calcium storage. The increased ER stress can also be attributed to chaperonin downregulation [68] and results in the activation of a signal transduction cascade (unfolded protein response, UPR) that promotes JUN N-terminal kinase (JNK), NF-ĸB, and caspase-12 activation [44].

### 2.4. Lipid Perioxidation

Steatotic livers are more prone to lipid perioxidation, following ischaemia and post-reperfusion [42,69,70]. Lipid perioxidation (oxidative degradation of lipids) is characterised by a reduction in hydrogen peroxide resulting in a hydroxyl radical involved in the destruction of polyunsaturated fats [71]. These free radicals scavenge electrons from lipids located in cell membranes and aggravate cellular injury [72]. In clinical LT, lipid perioxidation has been associated with oxidative injury induced by ROS during reperfusion [73].

### 2.5. Pro-Inflammatory Environment

Following implantation, the reperfusion of steatotic livers is associated with an exacerbated inflammatory response driven by pro-inflammatory mediators including TNF-α and neutrophils. Cytokine release is associated with endothelial dysfunction, increased expression of adhesion molecules and the activation and migration of platelets and leukocytes [74,75,76,77]. Kupffer cell activation results in sustained ET-1 production, lower phagocytic activity and increased ROS, IL-6, and IL-Iβ production in steatotic livers compared to lean livers [78,79]. The ongoing activation of inflammatory cells further promote ROS and protease production, thereby promoting hepatocellular injury [78].

### 2.6. Hypoxia Inducible Factors

Hypoxia has been attributed as a central instigator in the development and progression of hepatic steatosis due to the structural distortion caused by hepatocyte swelling and fibrotic scar formation, increased metabolic demands, oxygen consumption, and perturbation of lipid homeostasis [80]. Hypoxia-inducible factors (HIFs) are cellular oxygen sensitive transcription factors which have been implicated as the ‘master regulators’ in response to hypoxia through activation of a number of hypoxia responsive genes. The HIF-1α isoform has demonstrated a protective effect through a reduction in lipid synthesis, de novo lipogenesis, and lipid peroxidation, as well as promotion of fatty acid β-oxidation [81]. However, the HIF-2α isoform is reported to activate genes involved in fatty acid synthesis (Srebp1c and Fasn), fatty acid uptake (Cd36), and suppression of genes that regulate fatty acid β-oxidation, resulting in progression of lipid accumulation and fibrosis [80,82]. Pre-clinical murine studies have demonstrated the benefit of pre-treatment with HIF prolyl-hydroxylase inhibitors and other pharmacological agents including Mangafodipir (a contrast agent) in upregulation of HIF-1α expression with improved liver graft tolerance to IRI during reperfusion [83,84]. This effect has also been demonstrated in steatotic murine livers that received pre-treatment with trimetazidine (an anti-ischaemic drug) through activation of cytoprotective genes (heme-oxygenase associated with HIF-1α [84]. In addition, a recent study by Dery et al. [85] has demonstrated carcinoembryonic antigen-related cell adhesion molecule 1 (CEACAM1) alternative splicing, mediated by hypoxia-inducible factor 1 alpha (HIF-1α) in response to stress, significantly augmenting hepatic ischaemic tolerance in the hepatic tissues of both mice and humans. The research delineates the mechanism of action, demonstrating HIF-1α’s direct association with the promoter region of the polypyrimidine tract-binding protein 1 (PTBP1) splicing enzyme. This interaction facilitates the induction of the CEACAM1-short isoform, thereby enhancing ischaemic tolerance with a reduction in DAMPs including Histone H3 expression. Importantly, the findings from this study underscore a novel biomarker for assessing the viability of liver transplant donor livers.

### 2.7. Damage-Associated Molecular Patterns (DAMPs)

DAMPs typically originate from an intracellular source (cytoplasm, mitochondria, and nucleus) and are associated with hepatocellular injury during reperfusion [86,87,88,89]. More recently, attention has been drawn to DAMPs of nuclear origin that have a significant potential to exacerbate injury during liver IRI. Nuclear DAMPs include nucleosomes as well as free nucleic acids and proteins such as cell-free (cfDNA), mitochondrial DNA, free histones, and HMGB-1 that are released during unprogrammed cellular injury or death [90,91,92]. The interaction of these molecules with Toll-Like-Receptors (TLR), RAGE (Receptor for Advanced Glycation Endproducts) and pattern recognition receptors (PRRs) in the activation of innate immune and inflammatory responses [93,94,95] during reperfusion thereby propagates the magnitude of injury/inflammation, which is already heightened in steatotic livers [86,96].

### 2.8. Neutrophils and NETosis

Neutrophils in circulation are mobilised to the site of tissue injury where they interact with activated endothelial cells, facilitating their adhesion and subsequent migration into the sub-sinusoidal spaces. This migration is mediated by the interaction between integrin αMβ2 (Mac1) on neutrophils and Intercellular Adhesion Molecule 1 (ICAM-1) on liver sinusoidal endothelial cells (LSECs), a process that is directed by chemokines from Kupffer cells and a chemotactic gradient originating from the site of injury [97,98,99]. Upon reperfusion, neutrophils contribute to the exacerbation of tissue damage through the promotion of inflammatory responses [100]. A key mechanism through which neutrophils augment liver injury during IRI involves the release of NETs—structures composed of extracellular DNA decorated with histones and granular proteins. The phenomenon of NET formation, or NETosis, represents a recently elucidated mode of neutrophil death, distinct from the caspase-dependent pathways of apoptosis. Unlike apoptosis, which leads to the generation of apoptotic bodies without subsequent inflammation, NETosis encompasses both lytic and non-lytic pathways that culminate in the dissemination of nuclear contents into the extracellular milieu. The lytic pathway of NETosis is marked by the disintegration of the nuclear envelope, cellular depolarisation, chromatin de-condensation and cell membrane rupture leading to the dispersion of extracellular chromatin fragments into circulation. Conversely, the non-lytic pathway of NETosis does not culminate in cellular death but involves the expulsion of a mesh-like structure of decondensed chromatin, decorated with intracellular and granular proteins, into the extracellular space. The formation of NETs triggers several processes that intensify the severity of IRI, including thrombosis and the amplification of inflammatory responses resulting in liver graft injury [94,95,101].

The described mechanisms, while not yet fully elucidated, lay the foundational groundwork for devising strategies aimed at mitigating IRI injury in steatotic donor livers.

## 3. Static Cold Storage

The primary objective of liver preservation for transplantation is to reduce the magnitude of ischaemic injury (resulting from anaerobic metabolism and hypoxia) whilst maintaining structural and functional integrity [102,103]. Post-transplant liver function is related to the pathophysiological process of brainstem/cardiac death and subsequent ischaemic injury (related to organ retrieval and the preservation environment). Therefore, the optimisation of each stage of the donor pathway, from retrieval to implantation, is essential to ensure satisfactory outcomes in the recipient [104].

### 3.1. Principles of Cooling and Mechanisms of Injury

The current standard for liver preservation is SCS, this involves rapid cooling of the liver to 4 °C. This is achieved by flushing of the liver with cold specialised organ preservation solution and effectively removing residual blood from the liver until the effluent is clear and the liver is uniformly pale and cooled down. Subsequently, the liver is immersed and stored in preservation solution (0–4 °C), enclosed in sterile bags, and positioned on ice within an insulated organ transport ice box. At this reduced temperature, the liver’s metabolic rate is reduced to 10% of its normative rate at physiological body temperature. This reduction is facilitated by the application of organ preservation solutions (OPS), which prevent cellular swelling as the temperature drops and cease cellular membrane functions. Rapid execution of these steps, from the initial cold perfusion within the donor to the final packaging and placement in an ice box, is crucial to sufficiently lower the temperature, diminish metabolic rate, and preserve cellular energy stores [104].

OPS are designed to mitigate cellular alterations, such as the swelling and lysis that are evident during SCS. The depletion of ATP, increased glycolysis, and accumulation of lactic acid are all consequences of prolonged cold-ischemia time (CIT) in the context of SCS. Typically, the duration of CIT is restricted to less than 12 h, with even stricter limitations for grafts classified as high-risk, such as donation after circulatory death (DCD) grafts and those exhibiting steatosis [104]. These particular grafts are more vulnerable to hypoxia and the impacts of anaerobic metabolism, which include the production of metabolites that serve as precursors for IRI upon transplantation into the recipient [102,103].

**Figure 1:**
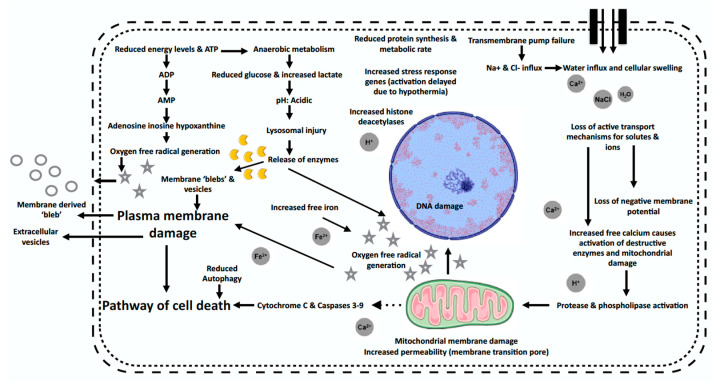
Pathophysiological mechanisms of cell injury and death occurring during cold preservation and reperfusion injury. Oxygen free radical generation results from the subsequent accumulation of ATP breakdown products including hypoxanthine. Cellular acidosis ensues and is characterised by an interruption of cellular mechanisms that are both energy and pH dependant. These include disruptions in function of transmembrane ion pumps (Na^+^/K^+^ and Ca^2+^) which are responsible for maintaining cellular integrity through regulation of intracellular ion composition. There is an influx of Na^+^, Cl^–^ and water with increased cell swelling and loss of membrane potential. Injury to the cell membrane is propagated by lysosomal enzymes (due to intracellular acidosis) and free radicals (due to oxidative injury). There is a rise in intracellular Ca^2+^ due to membrane pump failure causing activation of deleterious proteases and phospholipases. This initiates a cascade of mitochondrial membrane injury with release of cytochrome C with resultant apoptotic cell death. Overall, the mitochondrial dysfunction driven by acidosis, increasing intracellular Ca^2+^ and Fe^2+^ and lysosomal activation are significant contributors to the pro-oxidant milieu driving oxidative stress and reactive oxygen species (ROS) production at organ reperfusion. Figure modified from Fuller et al. [103]. Source: reproduced with permission from Elsevier [108].

A description of the biochemical properties and associated pathophysiological pathways [103,105] required for the development of OPS are described in Table 1 and Figure 1 [106]. Cooling serves as the primary mechanism for protecting against hypoxic damage by diminishing cellular metabolism and the need for oxygen. Nonetheless, even at 4 °C, metabolic activity continues, albeit at a reduced level, impacting several biochemical pathways. This includes the suppression of the Na^+^/K^+^ ATPase, leading to cellular oedema. The activity of mitochondrial enzymes is also decreased due to the cooling-induced hypothermia. The transport of adenosine diphosphate (ADP) into mitochondria, which is essential for the synthesis of ATP, relies on the membrane adenine nucleotide translocase. However, hypothermia impairs the function of this translocase, thereby decreasing the availability of ADP for ATP production and resulting in a net decrease in ATP. Early in this process, depolarisation of the cell membrane disrupts the balance of ions, contributing to cell death through either apoptosis or necrosis, in conjunction with other membrane and intracellular disturbances. Disruptions in calcium transport and an increase in anaerobic glycolysis lead to intracellular acidosis, ultimately causing mitochondrial dysfunction and fatal cellular damage. The principal factor causing organ damage during reperfusion is the generation of oxygen free radicals. Ischemia elevates intracellular Ca^2+^ levels, which activate the cytosolic enzymes that transform xanthine dehydrogenase into xanthine oxidase. This enzyme is involved in breaking down hypoxanthine and xanthine into uric acid and facilitates the production of superoxide by using molecular oxygen as an electron acceptor. This superoxide then reacts to produce hydrogen peroxide, a damaging oxidant that compromises lipid membranes and proteins. Subsequently a cascade of free radical reactions is initiated, including the generation of highly reactive singlet oxygen and hydroxyl radicals, which cause significant damage to the organ upon reperfusion [107].

OPS are designed to mitigate the damage to organs incurred during cold preservation. The primary objectives of these solutions include the following: (i) prevention of cellular swelling and the development of interstitial oedema as a result of hypothermia; (ii) maintaining electrolyte balance and preventing disturbances; (iii) prevention of cellular acidosis and aversion of cellular degeneration; (iv) protection against damage from oxidative stress; and (v) supplying the critical substrates necessary for cellular energy production (ATP generation) and enhancing organ function following reperfusion [109].

Hypothermia (0–4 °C) is critical for decreasing metabolic rates to a level that supports membrane integrity and essential cellular functions in an oxygen-deprived environment. Nevertheless, the combination of hypoxia and hypothermia during SCS leads to both interstitial and cellular oedema, marked by a fluid shift from the intravascular to the interstitial compartment. This shift can be mitigated by incorporating colloids into the preservation solution. Colloids are large molecules that are retained within the intravascular space, thereby creating an osmotic pressure that limits the movement of water into the interstitial space. Hydroxyethyl starch (HES), polyethylene glycol (PEG), dextran, and albumin are among the colloids frequently utilised for this purpose. Additionally, the inclusion of impermeant(s) in preservation solutions offers further benefits: after the livers are flushed and subsequently equilibrated with the OPS, impermeants remain in the interstitial space and limit the degree of cellular oedema. Key impermeants include various saccharides (such as raffinose, sucrose, mannitol, and glucose) and anions (such as lactobionate, gluconate, and citrate) [110].

During SCS, the shift towards anaerobic metabolism leads to an increase in intracellular H^+^ and lactate concentrations, culminating in cellular acidosis and subsequent cell death. To counteract this, the integration of a pH buffer into OPS is essential, with histidine and phosphate being among the commonly utilised buffers. Additionally, SCS is characterised by the generation of oxygen free radicals, which can cause hepatocellular injury, affecting nucleic acids, proteins, and lipids. To mitigate such oxidative damage, OPS are formulated with components that effectively neutralise oxygen free radicals. These include substances known for their antioxidant capabilities, such as tryptophan, mannitol, glutathione, and the inclusion of allopurinol [110].

### 3.2. Organ Preservation Solutions in Liver Transplantation

Collins et al. were pioneers in creating the first modern OPS [111] and utilised impermeants that maintained the osmotic equilibrium across the cell membrane which was otherwise compromised due to the failure of the Na^+^/K^+^ pump during SCS. With the addition of glucose as an impermeant, this formulation was subsequently named the Collins C2 solution [105]. Concurrently, Marshall et al. formulated a hyperosmolar citrate solution, incorporating mannitol as the impermeant. This formulation, known as Soltran (Baxter), was widely utilised in kidney transplantation until recently [112,113].

Since the 1980s, various OPS have been adopted for LT. The University of Wisconsin (UW) solution, developed by Belzer (Surgeon) and Southard (Scientist) stands out as a pioneering formulation. The UW solution is characterised by its high viscosity and an electrolyte profile resembling that of the intracellular environment (low Na^+^ and high K^+^) [114,115,116]. This OPS has enabled extended preservation times: Todo et al. reported successful LT after more than 15 h of SCS, significantly enhancing the feasibility of transporting organs over long distances and thus revolutionising LT logistics [117].

The UW solution, while building upon the foundational principles of earlier OPSs, introduced several innovative components: (i) allopurinol, to inhibit xanthine oxidase and offer antioxidant protection; (ii) adenosine, as an ATP precursor during liver reperfusion, supplemented with insulin to aid glycolysis; (iii) glutathione, to act as a free radical scavenger and provide antioxidant support; (iv) HES, a colloid with high molecular weight for preventing interstitial oedema; and (v) lactobionate and raffinose as impermeants to substitute for the more permeable Cl^−^ and glucose, thereby averting cellular swelling. In addition, potential additives include dexamethasone (for stabilising lysosomal membranes) and penicillin (to inhibit microbial growth) [118].

The principal components of UW solution that contribute significantly to liver preservation include the impermeant lactobionate, which not only prevents cellular swelling during storage but also acts as a chelator for Ca^2+^ and free Fe^3+^. Glutathione is considered another critical component, with studies indicating that its absence can lead to decreased survival rates of liver grafts post-transplantation. Although adenosine contributes to the efficacy of UW solution, its impact is less pronounced than that of lactobionate. It is also a cardioactive compound that can lead to transient bradyarrhythmias when flushed from the liver into circulation. For this reason (and due to the high K^+^ content of UW solution) the liver graft is often flushed, i.e., with saline or blood to remove any residual contents prior to implantation in the recipient [118].

Custodiol^®^ (HTK), formulated by German physiologist Bretschneider at the University of Göttingen in the early 1970s, was originally developed as a cardioplegia fluid and introduced into clinical heart transplantation in the 1980s [119]. The solution is characterised by its low K^+^ and high concentration of histidine (acting as buffer). The addition of histidine to the solution enhances the osmotic effect of mannitol and facilitates the maintenance of a physiological pH under conditions of anaerobic glycolysis and hypothermia. Additional components include tryptophan, which supports membrane integrity, and ketoglutarate, serving as an antioxidant. Due to HTK’s low viscosity (compared to UW solution), it is believed that a greater volume of infusion during organ retrieval is required to achieve comparable cooling. The results in a more rapid organ flush and its use has been advocated by several authors in the context of living donor or DCD LT due to the lower vascular resistance compared to livers flushed with the more viscous UW solution. In the 1990s, HTK was being successfully used for liver preservation in Europe and received FDA approval in the United States in 2002 [120]. However, the initial clinical trials and registry data indicate the superior performance of UW solution compared to HTK, thus establishing its status as the benchmark OPS for liver preservation [106,121]. The introduction of UW (alongside the advent of calcineurin-inhibitor based immunosuppression) has been pivotal in advancing the early success and evolution of LT.

Celsior^®^, initially developed as a cardiac preservation solution, utilises features of UW (inert osmotic agents) and HTK (robust buffering capacity, low viscosity) with an electrolyte composition similar to that of the extracellular compartment (high Na^+^ and low K^+^). Constituents of the solution include histidine, mannitol, and lower concentrations of glutathione [122,123]. Celsior^®^ received approval from the Food and Drug Administration (FDA) in the United States for cardiac preservation in 1999 [109].

IGL-1^®^, a newer preservation solution, has shown outcomes comparable to those of UW and HTK solutions, with additional advantages in preserving steatotic liver grafts observed in experimental studies [109]. The composition of IGL-1^®^ is principally based on UW solution with biochemical composition that reflects the extracellular compartment; i.e., high Na^+^ and low K^+^ with the addition of PEG, a colloid with lower viscosity compared to HES [124,125,126]. The advantageous effects of PEG during cold preservation can be attributed to a reduction in shear stress and enhanced microcirculation, a result of the lower viscosity. Specifically, substituting HES with PEG significantly decreases the viscosity of IGL-1^®^ compared to UW (1.28 vs. 5.7 millipascal-second). The reduction in viscosity is correlated with enhanced cellular protection mechanisms and a reduction in mitochondrial damage resulting from the activation of hepatoprotective pathways including adenosine monophosphate-activated protein kinase (AMPK) and endothelial NO synthase (eNOS) [127]. These mechanisms have been implicated in recent studies of human hepatocytes subjected to IRI in vitro [128].

### 3.3. Organ Preservation Solutions and Steatotic Donor Livers

In the context of steatotic liver preservation with OPS, most studies comparing the efficacy of different OPS have been performed in pre-clinical experimental settings. In 2006, Mosbah et al. demonstrated IGL-1^®^’s enhanced utility in the preservation of steatotic rat livers [129]. Compared to UW, livers preserved with IGL-1^®^ demonstrated lower transaminase release, improved bile production, reduced levels of malondialdehyde (MDA, indicative of lipid peroxidation and oxidative damage), decreased activity of glutamate dehydrogenase (GLDH, indicative of mitochondrial damage), and reduced vascular resistance. The study also implicated the hepatoprotective effect of IGL-1^®^ against IRI through nitric oxide (NO), this was demonstrated by the increased expression of endothelial NO synthase (eNOS) in the IGL-1^®^ group and suppression of eNOS when a NO-inhibitor was added to the preservation solution.

Further investigations by the same group into the mechanisms behind IGL-1^®^’s enhanced preservation effects on steatotic livers demonstrated that enriching IGL-1^®^ with insulin-like growth factor-1 or epidermal growth factor enhanced eNOS activation and the hepatoprotective capacity against IRI [130,131]. Elevated levels of HIF-1α were observed in livers preserved with IGL-1, with increased expression of HO-1 (a gene downstream of HIF-1α) demonstrating the cytoprotective effect of this pathway [132]. Additionally, the anti-ischemic medication trimetazidine was found to elevate HIF-1α and sirtuin 1 levels while reducing HMGB1 concentrations, promoting autophagy to alleviate IRI [133].

In a study comparing IGL-1^®^ to Celsior^®^, Tabka et al. demonstrated similar results to Mosbah et al. [134]. Rat livers preserved with IGL-1^®^ demonstrated higher levels of endothelial NO synthase (eNOS) and a decreased activation of the pro-apoptotic mitogen-activated protein kinase (MAPK) pathway. The study demonstrated that arterial relaxation during preservation with IGL-1^®^ significantly relied on NO levels, supporting the hypothesis that IGL-1^®^ could mitigate endothelial dysfunction by activating eNOS. The addition of bortezomib (a proteasome inhibitor) facilitated the activation of adenosine monophosphate-activated protein kinase (AMPK) and subsequent upregulation of eNOS and glycogen synthase kinase 3 beta (GSK3β), which collectively contributed to diminished hepatocellular injury, oxidative stress, and apoptosis [135]. Furthermore, the inclusion of carbonic anhydrase II, a key enzyme in various processes related to IRI, enhanced the ability of IGL-1^®^ to activate AMPK. This activation effectively reduced UPR- and MAPK-related events resulting in improved liver function and histological outcomes [136]. These findings collectively affirm that the advantages of using IGL-1^®^ for the preservation of steatotic livers are closely linked to the activation of AMPK and eNOS [137,138].

The subsequent literature [125,139,140], in addition to substantiating the superiority of IGL-1^®^ compared to UW and HTK, demonstrated that the hepatoprotective effects of IGL-1^®^ in preserving steatotic livers are associated with the inhibition of proteasomes [140], the increased expression of aldehyde dehydrogenase 2 (ALDH2) [141], and the initiation of autophagy [142].

Similar to the experimental IGL-1^®^ studies, comparable improvements in eNOS induction and AMPK pathway activation during SCS have been demonstrated using UW solution. Supplementation of UW with pharmacological agents such as trimetazidine, aminoimidazole-4-carboxamide ribonucleoside, carvedilol, or bortezomib during SCS of steatotic rat livers was characterised by a reduction in perfusate transaminases, increased bile secretion, reduced vascular resistance, and reduced malondialdehyde (MDA) and glutamate dehydrogenase (GLDH) expression during reperfusion [70,143,144,145].

Eipel et al. investigated the impact of supplementing erythropoietin (EPO) to HTK solution during SCS of steatotic rat livers. The authors demonstrated increased oxygen utilisation, improved endothelial integrity, and a minor decrease in AST levels following reperfusion. Nonetheless, EPO supplementation did not affect UCP-2 expression, and the underlying mechanisms for the observed preservation benefits remain subject to further investigation [146].

To further diminish ROS production and mitigate hepatocellular damage, a novel IGL solution (IGL-2) has been formulated. ILG-2 contains an increased concentration of PEG (5 vs. 1 g/L) and glutathione, with histidine and mannitol introduced as impermeants instead of raffinose [147]. The preservation of steatotic livers with IGL-2 demonstrated a reduction in mitochondrial damage and oxidative stress, indicated by elevated levels of HO-1, glutathione, ALDH2, and mitochondrial complexes I and II (thereby ameliorating IRI) [147,148,149]. Notably, livers preserved with IGL-2 exhibited the least water retention during preservation, suggesting that PEG may help in reducing interstitial swelling.

The outlined experimental data indicate that OPS with PEG offer benefits for maintaining mitochondrial integrity and preventing oxidative damage. Among these, IGL-1^®^ and IGL-2 emerge as the most suitable options for the SCS of steatotic livers. Nonetheless, these outcomes should be approached with caution due to the absence of data from the setting of clinical LT. Moreover, IGL-2 has not yet received clinical approval, and the advantage of PEG-enriched solutions needs validation through clinical research.

## 4. Normothermic Machine Perfusion

### 4.1. Normothermic Machine Perfusion

The imperative to broaden the utilisation of donor organs (particularly those of the high-risk category) and reduce waiting list mortality rates has played a pivotal role in the evolution of NMP technology. NMP, which utilises a blood-based perfusate (packed red blood cells, pRBCs) warmed to physiological temperature (37 °C) enriched with oxygen and nutrients, is designed to keep the donor liver in a functional, near-physiological condition prior to LT. NMP can be applied in a continuous manner, i.e., at the point of organ retrieval (device-to-donor or continuous NMP, cNMP) or after a period of SCS (back-to-base, end-ischaemic or post-SCS NMP). The benefits attributed to NMP in comparison to traditional preservation techniques encompass the following: (i) the capability to recover from acute injury (such as hypoxia) incurred before or during the organ retrieval process, particularly in DCD organs; (ii) the ability to objectively evaluate liver functionality prior to transplantation (through the assessment of metabolic or synthetic activities), facilitating the identification of high-risk organs that would otherwise be deemed unsuitable for transplantation (with criteria established through clinical research); (iii) enhancement of transplant logistics via prolonged preservation durations; (iv) restoration of ATP levels and modulation of apoptosis, immune responses, and enhancement of regenerative pathways; and (v) the prospect of administering therapeutic treatments to the donor liver prior to transplantation (as indicated by experimental research) [150,151,152,153,154].

A major advantage of NMP over SCS and other dynamic preservation methods is its capability to evaluate liver function parameters during perfusion. This enables the assessment of liver viability prior to exposing a recipient to the risks associated with LT. Researchers in Cambridge and Birmingham have identified criteria including metabolic indicators (such as lactate clearance), markers of organ injury (like perfusate transaminase levels), hemodynamic measurements (such as perfusion flow rates), and indicators of potential biliary complications (bile biochemistry), detailed in Table 2 and Table 3 [155,156]. Additionally, the team in Groningen has identified histological indicators of bile duct injury (BDI) during NMP with a BDI scoring system (ranging from 0 to 7) based on stroma necrosis, damage or loss of extramural peribiliary glands, and vascular lesions. High BDI scores have been linked to poor biliary function predictors, including low bile pH and high levels of glucose and lactate dehydrogenase (LDH) [157].

Initial evidence regarding the utility of NMP emerged from case studies in Birmingham [158] and Cambridge [159]. The safety and efficacy of continuous NMP (cNMP) was first demonstrated by Ravikumar et al. from Oxford [160], confirming the feasibility of this preservation technique, but also demonstrating a substantial reduction in graft injury compared to SCS. The landmark multicentre randomised controlled trial (RCT) involving 220 liver transplant recipients, was carried out by Nasralla et al. in 2018 under the Consortium for Organ Preservation in Europe (COPE), comparing cNMP with SCS. NMP-treated livers showed a notable reduction in reperfusion-related injury, evidenced by a 49% decrease in peak post-operative AST levels, and a 50% reduction in the rate of organ discard, despite a 54% extension in mean preservation duration. The enhanced rate of organ utilisation, marked by the lower discard rate among cNMP-treated livers, was partly credited to the capacity for direct functional assessment of liver function for viability, thus offering surgeons increased confidence in utilising higher-risk donor livers [161]. In a subset analysis of steatotic donor livers from this cohort, Ceresa et al. explored the impact of cNMP on histologically confirmed steatotic livers with matched lean controls preserved with cNMP. Steatotic livers demonstrated distinct variations in lipid metabolism during perfusion compared to lean livers, characterised by enhanced triglyceride mobilisation, increased mitochondrial fatty acid β-oxidation and greater hepatocellular injury (quantified by perfusate transaminase levels). Nonetheless, steatotic livers preserved with cNMP had comparable outcomes to lean livers preserved with SCS, suggesting a reduction in risk profile of these livers facilitated by enhanced preservation during cNMP. In addition, when compared to steatotic livers with SCS there was no significant histological reduction in the degree of macrovesicular steatosis (MaS) during cNMP [162].

In 2021, Markmann et al., representing the OCS Liver Protect Randomized Clinical Trial Group in the United States, shared findings from a multicentre RCT examining the impact of cNMP on transplant outcomes. This RCT compared NMP with SCS in 293 patients (per protocol population). Unlike the COPE trial, the PROTECT trial featured shorter average NMP durations, with a mean (SD) of 276.6 (117.4) minutes. The study highlighted that NMP was associated with a reduction in EAD and ischaemic bile duct complications (IBC), fewer histological features of IRI, reduction in ICU and hospital stay, improved utilisation of DCD livers, and graft survival. The research design limited inclusion to livers with MaS of 40% or below, but it did not clearly detail the actual steatosis levels of the included livers [163].

Recently, Webb et al. demonstrated the cost-effectiveness of NMP in the Canadian LT setting. A Markov model was used to evaluate the cost-effectiveness of integrating NMP with SCS vs. SCS alone (control) in LT, analysing strategies over five years from a public healthcare payer’s view. Cost data derived from a single-centre retrospective trial and supplemented by literature-based utility values and transition probabilities demonstrated that NMP was cost-effective, showing a lower mean cost (USD 456,455 vs. USD 519,222) and higher quality-adjusted life years (QALYs) gains (3.48 vs. 3.17) compared to the control strategy. The robustness of these findings was confirmed through scenario and probabilistic sensitivity analysis, where NMP proved cost-effective in 63% of iterations at a willingness-to-pay threshold of USD 40,941, supporting its beneficial addition to liver transplant programs [164].

The logistically more straightforward approach of conventional SCS, followed by connection of the donor liver to the normothermic perfusion device at the recipient centre (back-to-base), has also been explored by Ceresa et al. who reported a series of liver transplants preserved with pSCS-NMP. Adhering to the same recruitment criteria as a COPE trial, a total of 31 livers were transplanted using the back-to-base (pSCS-NMP) approach with no significant differences in graft injury indicators (peak AST in the first 7 post-operative days), graft and patient survival, or organ discard rates when compared to a matched selection of cNMP from the COPE trial [165]. However, out of the 31 livers included in the pSCS-NMP cohort, 3 (9.7%) were discarded. Of the discarded livers, one liver had histological evidence of 80% MaS with poor lactate clearance, glucose metabolism and lack of bile production during perfusion.

In the recent VITTAL study by the Birmingham group, previously declined livers were perfused, and those meeting pre-defined functional criteria were transplanted: in all, 22 of 31 (71%) perfused organs were transplanted, all with immediate function. Notably, the livers that did not meet viability criteria tended to be heavier with increased donor peak AST, longer CIT (550 vs. 452 min), and a greater proportion (77.8% vs. 40.9%) had MaS > 30% [156].

Patrono et al. further investigated the utilisation and outcomes of steatotic donor livers preserved with pSCS-NMP and MaS ≥ 30%: a total of 10 out of 14 livers (71.4%) were transplanted, of which 2 livers (14%) developed PNF. This study highlights the challenges in determining the viability of livers with moderate-severe steatosis using existing viability criteria. The results indicate that an extended observation period (≥6 h) may be necessary for these livers, emphasising that consistent lactate clearance is essential for their utilisation [166]. To address the limitation of pSCS-NMP of steatotic donor livers, Patrono et al. further published a proof-of-concept case utilising device-to-donor (cNMP) on a HCV-positive DBD donor liver with 70% MaS. This case met the functional assessment benchmarks described by both the Birmingham and Groningen groups and was transplanted with 6-month graft and patient survival, normal post-operative graft function and the absence of any clinical or laboratory signs of ischemic cholangiopathy [167].

Whilst pSCS-NMP has several advantages, this end-ischaemic approach may result in suboptimal results and discards of steatotic livers that are sensitive to even short periods of CIT, and this is evident in aforementioned end-ischaemic (pSCS-NMP) studies that have included a small proportion of steatotic livers within the overall cohort. These data suggest steatotic donor livers may require active intervention beyond that of simply replacing SCS with NMP.

More recently, He et al. explored the advantages of preventing ischaemia and complete avoidance of cooling prior to LT, i.e., ischemia-free liver transplantation (IFLT) which involves the procurement, preservation, and transplantation of the donor liver using continuous normothermic perfusion without interruption of blood flow. The authors described the successful transplantation of a steatotic DBD liver with 85–95% MaS [168]. The clinical application of IFLT has been further described by Chen et al. in a study that included 26 steatotic livers (16 with moderate and 10 with severe steatosis). Within this cohort, six livers (23.1%) underwent IFLT and demonstrated a reduction in peak AST, GGT, and creatinine post-transplant with a significant reduction in EAD (0% vs. 60%; *p* = 0.001) [169]. These findings have also been further validated in a randomised clinical trial by Guo et al. [170]. Nonetheless, to fully assess the potential of NMP to enhance the viability of steatotic donor livers for transplantation, a comparative analysis with livers preserved via post-SCS-NMP, cNMP, and IFLT is required.

In the current clinical setting, IFLT is a sophisticated technique carried out primarily in high-volume, experienced centres in China, making it less accessible globally. Continuous cNMP emerges as a practical alternative, navigating between the constraints of end-ischaemic NMP (pSCS-NMP) and the advantages of IFLT for the preservation of steatotic donor livers. Crucially, in the current era where IFLT is not widely available, ex situ preservation (including pSCS-NMP and cNMP) may offer a platform for therapeutic interventions such as defatting therapies that target hepatocellular lipid metabolism during NMP as an alternative strategy to improve ex situ function and the risk profile of livers with moderate-severe steatosis.

### 4.2. Normothermic Perfusion as a Therapeutic Platform for Steatotic Donor Liver Optimisation

Preclinical studies have demonstrated that NMP can improve ex situ liver function and reduce intrahepatic triglyceride content (IHTG) through the promotion of lipid metabolism and thereby potentiating the reversal of steatosis. Jamieson et al. [171] investigated the effect of NMP alone in steatotic porcine livers preserved over 48 h. In this model, HS was induced through pre-treatment with streptozotocin and a high-fat diet, inducing hyperglycaemia and ketosis prior to organ retrieval for NMP. During perfusion, steatotic livers maintained perfusate base excess, factor V and bile production. In addition, the perfusion haemodynamics and hepatocellular injury markers were comparable to that of the lean controls. Notably, these livers demonstrated elevated levels of glucose and urea in the perfusate. Following 48 h of perfusion, there was a significant reduction in MaS from 28% to 15% and the size of lipid droplets, achieved without the use of defatting agents.

Nagarth et al. [172] developed an experimental oxygenated normothermic model to study the impact of a ‘defatting cocktail’ on steatotic livers retrieved from Zucker rats during 3 h perfusions. This cocktail was a combination of pharmacological compounds, including GW501516 (a PPARδ ligand), GW7647 (a PPARα ligand), forskolin (a cAMP activator), hypericin (a pregnane X receptor ligand), visfatin (an insulin-mimicking adipokine), and scorparone (a constitutive androstane receptor ligand), (see Table 4). Following addition of the ‘defatting cocktail’ to the NMP perfusate, there was a 65% decrease in hepatic triglyceride levels and a 50% reduction in intracellular lipid content. In contrast, livers perfused without the ‘defatting cocktail’ demonstrated a 30% reduction in hepatic triglycerides [172]. Raigani et al. [173] demonstrated comparable outcomes by incorporating L-carnitine (to enhance fatty acid β-oxidation) as a constituent of the ‘defatting cocktail’. The interventions led to a reduction in MaS from 41.5% to 8.5%. Furthermore, there was a rise in ketone levels in the perfusate (indicative of enhanced fatty acid β-oxidation), as well as increased bile bicarbonate content and improved lactate clearance in the steatotic rat livers that received these interventions.

These preclinical studies demonstrate the potential of NMP as a platform to provide active intervention to treat donor HS. The findings indicate that both hepatic triglyceride levels and MaS can be altered during ex situ preservation. However, the limited sample size and uniformity of the livers, where steatosis was experimentally induced, suggest caution when extrapolating these results to clinical settings involving a heterogenous group of steatotic human livers intended for transplantation. The effectiveness of defatting agents in these animal models presents mixed outcomes; NMP by itself has demonstrated the capacity to lower hepatic triglyceride levels and enhance liver function to levels seen in lean counterparts.

The effect of defatting agents during NMP would be better investigated in a discarded steatotic human liver model. Initial results from a discarded human liver study involving NMP of steatotic donor livers for 24 h did not demonstrate an overall reduction in MaS [174]. However, Banan et al. reported outcomes from two human livers preserved with NMP and defatting agents (L-carnitine and exendin-4), with one liver demonstrating a 10% decrease in MaS after 8 h of NMP [175].

More recently, Boteon et al. [176] explored the use of the ‘defatting cocktail’ developed by Nagarth et al. [172] with the addition of L-carnitine in livers declined for transplantation due to advanced steatosis. These perfusion experiments included a total of 10 steatotic human livers perfused with either the modified ‘defatting cocktail’ during NMP (*n* = 5) and with NMP alone (*n* = 5). Discarded livers that were subjected to pharmacological defatting during NMP demonstrated improvement in metabolic function, decreased vascular resistance, reduction in hepatocellular injury and greater bile production. Mechanistic studies also demonstrated a reduction in oxidative damage, immune cell activation, inflammatory cytokine release and tissue triglycerides, achieving a 40% reduction in MaS after 6 h of perfusion.

In addition, all five livers that received pharmacological defatting during NMP met viability criteria for transplantation, in contrast to only two out of five in the control group (*p* = 0.04). However, not all defatted livers were able to demonstrate a clinically meaningful reduction in MaS of <30%. This raises questions about the relationship between histological steatosis and liver function during NMP. Mechanistically, it is possible that cytoprotective and vaso-protective pathways are important factors that render such organs suitable for transplantation and that NMP and defatting may have synergistic effects in achieving ex situ functional criteria for transplantation [177].

Although Boteon et al. demonstrated favourable outcomes, the pre-clinical nature of the study did not involve actual liver transplants. A thorough assessment of the safety profile for the suggested ‘defatting cocktail’ is essential before it can be adopted in clinical settings. Many components of the ‘defatting cocktail’ are yet to be fully evaluated for safety, despite some in vitro tests on cytotoxicity [78]. Hypericin, found in St John’s Wort, plays a role in enhancing the activity of the cytochrome P450 3A4 enzyme, which is crucial in metabolising drugs like cyclosporine and tacrolimus [178]. Furthermore, the peroxisome proliferator-activated receptor agonists, GW501516 and GW7647, have yet to undergo human trials, with existing animal studies raising concerns about potential carcinogenic effects [179].

Ceresa et al. [180] have recently described outcomes of a study involving 18 human discarded steatotic livers subjected to 48 h of NMP. Whilst designing the study, the authors addressed some of the limitations of previous defatting studies with consideration of requirements of translation into a subsequent clinical trial. This included use of readily available pharmacological agents, licenced for human use and with avoidance of pharmacological agents that would require extensive testing and optimisation prior to use in the setting a clinical trial. The study involved three groups: NMP alone (*n* = 6), NMP plus a lipid apheresis filter (*n* = 6), and NMP with a lipid apheresis filter and the adjunct of defatting agents including l-carnitine, water soluble forskolin (NKH-477), and glucose/insulin reduction (to reduce de novo lipogenesis), (*n* = 6). The use of the apheresis filter led to lowered triglyceride and cholesterol levels in the perfusate. Incorporating defatting agents promoted fatty acid β-oxidation and resulted in decreased steatosis, as evidenced by tissue triglyceride measurements. Whilst none of these livers were transplanted, structural and functional improvements were evident following 6 h of perfusion. These improvements, reflected in enhanced perfusion and biochemical parameters that would have rendered these livers transplantable based on current functional criteria, suggest that these organs could meet the current criteria for transplantation. The results suggest a minimum of 6 h of perfusion is necessary for functional assessment in order to assess suitability for transplantation.

Translating this earlier research, Abbas et al. [181] are currently running a blinded multicentre UK-based randomised clinical trial comparing the defatting protocol described by Ceresa et al. [180] with NMP alone in steatotic donor livers offered for transplant. The primary outcome of this clinical trial is the proportion of livers that meet all functional criteria for transplantation at 6 h of perfusion (described in Table 5). Secondary endpoints include the proportion of livers transplanted, post-operative graft function, evidence of IRI, ITU and hospital stay, non-anastomotic biliary stricture, and steatosis recurrence (determined by protocol MRI at 6 months) and graft/patient survival [181].

Abbas et al. [181] have also reported preliminary findings of dosing experiments using the defatting NMP protocol described by Ceresa et al. [180] with the adjunct of HIF modulators aiming at further attempts in reducing histological steatosis during perfusion in a series of discarded steatotic human livers. In this preliminary report, selective HIF-1α was achieved using deferoxamine (DFO, a potent activator of both HIF-1α and & HIF-2α) with selective HIF-2α inhibition using PT2385 (a HIF-2α dimerisation inhibitor). The authors demonstrated an accelerated reduction in histological MaS over 24 h perfusion [182]. In a series of DCD porcine perfusions, the same group has investigated alternative strategies to mitigate reperfusion injury and have reported use of a novel column (NucleoCapture) designed to remove DAMPs from the perfusate during perfusion. The model involved two phases: NMP with leucodepleted blood and simulated transplantation (ex situ) using whole blood for reperfusion. The authors were able to demonstrate improved lactate clearance, maintenance of hepatocellular function and importantly an overall reduction in circulating extracellular histones when the column was applied either during NMP or during whole blood reperfusion (following an initial 6 h of NMP alone) [182]. Whilst this technology has not been tested in discarded steatotic human livers, it holds promise in protecting high-risk grafts from IRI.

Further research on discarded steatotic human livers has been explored by Da Silva et al. [183] who have recently published on defatting ex situ over multi-day perfusions. In this study, a total of 51 liver grafts were included (23 discarded liver grafts and 28 partial livers obtained hepatic resections) and were subjected to NMP. Out of 51 liver grafts, 20 were steatotic with MaS up to 85% and were subjected to NMP for as long as 12 days. Of these, half showed significant reduction in steatosis and remaining half showed no change. The authors attributed defatting as a consequence of extended perfusion duration, regulated glucose levels, tailored nutrition, and supplementation with l-carnitine and fenofibrate. The majority of liver grafts maintained their synthetic and metabolic functions throughout the perfusion period.

## 5. Conclusions

The evolving landscape of LT is significantly challenged by the rising prevalence of liver diseases and the concomitant scarcity of suitable donor organs. This is further compounded by the global obesity epidemic driving MASLD (resulting in MASH) to the forefront of LT indications and at the same time also making HS more prevalent in the donor pool. The impact of HS on graft viability remains a concern, particularly for donor livers with moderate to severe steatosis which are highly sensitive to the process of IRI and SCS leading to poor post-transplantation outcomes.

In response to these challenges, the transplant community has been prompted to expand the criteria for donor organ selection, incorporating organs from DCD donors, those over the age of 65 and with evidence of moderate to severe steatosis. The adoption of the 2021 Banff consensus recommendations for the classification of donor HS represents a significant step towards standardising the assessment of HS, aiming to mitigate its impact on LT outcomes. Despite these advancements, innovative preservation strategies are required to address the heightened sensitivity of steatotic livers to IRI and the limitations of SCS.

The advent of NMP has emerged as a promising strategy to improve outcomes of steatotic liver grafts. NMP offers a dynamic preservation method that not only allows for the assessment of liver function prior to transplantation but also provides a platform for therapeutic interventions aimed at mitigating IRI and addressing HS. The ability of NMP to restore ATP levels, modulate apoptosis and immune responses, and enhance regenerative pathways holds significant potential to improve the outcomes of LT involving steatotic livers.

The clinical application of NMP has demonstrated notable successes, including enhanced organ utilisation rates, and the ability to evaluate the viability of high-risk donor livers for transplantation. Moreover, the potential of NMP as a therapeutic platform for pharmacological defatting and strategies to reduce reperfusion-related injury demonstrates its potential to increase utilisation of steatotic donor livers. However, the journey from experimental models to clinical practice is fraught with complexities. The safety profile of ‘defatting cocktails’, the efficacy of therapeutic interventions during NMP, and the translation of findings from preclinical studies to human trials necessitate careful consideration and rigorous evaluation.

The promising results obtained from studies involving discarded steatotic human livers and the ongoing clinical trials aiming to validate these approaches provide a glimpse into the future of LT, where the integration of advanced preservation technologies and targeted therapies could significantly expand the donor pool and enhance recipient outcomes. As the transplant community moves forward, the integration of scientific innovation with clinical practice will be paramount in overcoming the barriers to successful LT of livers of the high-risk category and ensuring that patients with end-stage liver disease have access to the life-saving treatment they desperately need.

## Figures and Tables

**Table 1:** Biochemical properties of organ preservation solutions to reduce cellular injury during static cold storage (SCS) [103,105,106]. Source: reproduced with permission from Elsevier [108].

Biochemical Property	Function
Colloids and impermeants	Prevent cellular swelling, counteract electrolyte and water movement through the cell membrane
Buffers	Stabilise the extracellular pH, deranged due to metabolites such as lactic acid during anaerobic metabolism
Antioxidants	Scavenge free radicals
Nutrients	Provide essential precursors for ATP production

**Table 2:** Cambridge NMP criteria for optimal NMP parameters associated with favourable post-transplant outcomes [155].

NMP Parameter	Description
Perfusate pH	The ability to maintain perfusate pH > 7.2 (without bicarbonate supplementation exceeding >30 mmol)
Bile pH	A maximum bile pH value > 7.5
Clearance of perfusate lactate	Evidence of a peak reduction in perfusate lactate ≥4.4 mmol/L/kg/h
Metabolism of glucose (perfusate)	Evidence of a reduction in perfusate glucose following 2 h of perfusion OR a glucose value of 10 mmol/L (that also subsequently falls following a challenge 2.5 g of glucose)
Bile glucose concentration	A bile glucose concentration of ≤3 mmol/L OR ≥10 mmol/L less than the perfusate glucose concentration
Hepatocellular injury as demonstrated by perfusate alanine aminotransferase (ALT) level	A perfusate ALT < 6000 IU/L at 2 h of perfusion

**Table 3:** Birmingham NMP criteria as recommended in the ‘VITTAL’ trial [156].

Mandatory NMP Parameter	Description
Clearance of perfusate lactate	Evidence of reduction in perfusate lactate ≤2.5 mmol/L
Two or more of the following NMP parameters at 4 h of perfusion	
Perfusate pH	The ability to maintain perfusate pH ≥ 7.3
Glucose	Evidence of glucose metabolism during perfusion
Bile	Evidence of bile production during perfusion
Vascular flows, i.e., hepatic arterial (HA) flow and portal venous (PV) flow	Maintenance of HA flow ≥150 mL/min and PV flow ≥500 mL/min
Macroscopic assessment of donor liver during NMP	Homogenous macroscopic appearance of the liver parenchyma during perfusion

**Table 4:** Constituents of ‘defatting cocktail’ described by Nagarth et al. [172].

Defatting Agent	Mechanism of Action
PPARδ ligand GW501516	A PPARδ ligand that improves fatty acid β-oxidation
Peroxisome proliferator-activated receptor (PPAR) α ligand GW7647	A PPARα ligand improves mitochondrial fatty acid oxidation
Forskolin, cyclic adenosine monophosphate (cAMP) activator (glucagon mimetic)	A cAMP activator that increases lipolysis and improves fatty acid oxidation
Hypericin	A pregnane X receptor ligand that increases β-oxidation of very long chain fatty acids
Visfatin	An adipokine (insulin-memetic, role not completely understood)
Scorparone	Upregulates PPAR (androstane receptor ligand)

**Table 5:** The defat study functional criteria for transplantation (at 6 h of perfusion) [181].

NMP Parameter	Description
Perfusate pH	The ability to maintain perfusate pH > 7.2
Bile pH	A minimum bile pH value > 7.5 (if bile produced)
Clearance of perfusate lactate	Clearance of lactate to a level <2.5 mmol/L
Metabolism of glucose (perfusate)	Evidence of a reduction in perfusate glucose (spontaneous fall)
Bile glucose concentration	A bile glucose concentration of ≤3 mmol/L OR ≥10 mmol/L lower than the perfusate glucose concentration
Hepatic arterial (HA) flow and portal venous (PV) flow	Maintenance of HA flow ≥100 mL/min and PV flow ≥500 mL/min
Hepatocellular injury as demonstrated by perfusate alanine aminotransferase (ALT) level	A perfusate ALT < 6000 IU/L at 6 h of perfusion
